# Prevalence and treatment of diabetes and pre-diabetes in a real-world heart failure population: a single-centre cross-sectional study

**DOI:** 10.1136/openhrt-2022-002133

**Published:** 2022-12-13

**Authors:** Erik Håkansson, Mattias Brunström, Helena Norberg, Sara Själander, Krister Lindmark

**Affiliations:** 1 Department of Public Health and Clinical Medicine, Umeå University, Umea, Sweden; 2 Department of Integrative Medical Biology, Umeå universitet Medicinska fakulteten, Umea, Sweden; 3 Department of Clinical Sciences Danderyd Hospital, Karolinska Institute, Danderyd, Sweden

**Keywords:** HEART FAILURE, Diabetes Mellitus, Epidemiology

## Abstract

**Aims:**

The aim of this study was to investigate a real-world heart failure (HF) cohort regarding (1) prevalence of known diabetes mellitus (DM), undiagnosed DM and pre-diabetes, (2) if hf treatment differs depending on glycaemic status and (3) if treatment of DM differs depending on HF phenotype.

**Methods:**

All patients who had received a diagnosis of HF at Umeå University Hospital between 2010 and 2019 were identified and data were extracted from patient files according to a prespecified protocol containing parameters for clinical characteristics, including echocardiogram results, comorbidities, fasting plasma glucose (FPG) and hemoglobin A1c (HbA1c) values. Patients’ HF phenotype was determined using the latest available echocardiogram. The number of patients with previous DM diagnosis was assessed. Patients without a previous diagnosis of DM were classified as non-DM, pre-diabetes or probable DM according to FPG and HbA1c levels using WHO criteria.

**Results:**

In total, 2326 patients (59% male, mean age 76±13 years) with HF and at least one echocardiogram were assessed. Of these, 617 (27%) patients had a previous diagnosis of DM. Of the 1709 patients without a previous diagnosis of DM, 1092 (67%) patients had either an FPG or HbA1c recorded, of which 441 (41%) met criteria for pre-diabetes and 97 (9%) met criteria for probable diabetes, corresponding to 19% and 4% of the entire cohort, respectively. Patients with HF and diabetes were more often treated with diuretics and beta blockers compared with non-DM patients (64% vs 42%, p<0.001 and 88% vs 83%, p<0.001, respectively). There was no difference in DM treatment between HF phenotypes.

**Conclusions:**

DM and pre-diabetes are common in this HF population with 50% of patients having either known DM, probable DM or pre-diabetes. Patients with HF and DM are more often treated with common HF medications. HF phenotype did not affect choice of DM therapy.

**WHAT IS ALREADY KNOWN ON THIS TOPIC**

- Heart failure (HF) and diabetes mellitus or pre-diabetes are commonly coexisting disorders which negatively impact patient outcome.
- Prevalence estimates for glycaemic disorders in HF patients differ between settings, and often come from selected populations, for example, trial participants.

**WHAT THIS STUDY ADDS**

- This single-centre study encompassing an unselected HF population finds that 27% of patients had known diabetes mellitus, 4% of patients had probable undiagnosed diabetes and 19% of patients had pre-diabetes.

**HOW THIS STUDY MIGHT AFFECT RESEARCH, PRACTICE OR POLICY**

- The high prevalence of glycaemic disorders warrants a generous approach to glycaemic testing in HF patients, and further studies on how to best manage pre-diabetes in HF.

## Background

Heart failure (HF) and diabetes mellitus (DM) are two common disorders that individually increase mortality, morbidity and healthcare costs.^1 2^ Patients with both conditions have a worse prognosis than patients with either diagnosis alone.^3^ The prevalence of DM in HF is high, between 20% and 40%, depending on geographical region and population studied, with the higher numbers seen in hospitalised patients.^4 5^ DM is increasingly viewed, not only as a comorbidity to HF, but also as a driver of HF, especially HF with preserved ejection fraction (HFpEF).^6^

Pre-diabetes, defined as above normal blood glucose and below the diagnostic cut-off for DM,^7 8^ is also associated with worse outcomes in HF.^9^ The prevalence of pre-diabetes has been less thoroughly studied than DM, and is mostly based on clinical trials for HF medications.^10–12^ Some patients are also first diagnosed with DM at inclusion in clinical trials.^13 14^ However, patient populations in clinical trials are often not representative of the population at large.^15 16^ There is a need for updated data on the prevalence of pre-diabetes and diabetes in modern HF populations.

The aim of this study was to investigate a Northern European real-world HF population regarding:

- Prevalence of pre-diabetes, undiagnosed DM and known DM.
- If HF treatment differs depending on DM status.
- If DM treatment differs depending on HF status.

## Methods

### Study population

We identified all patients who had received a diagnosis of HF (10h revision of the International Classification of Disease and Related Health Problems codes I50.X, I42.0, I46.6, I42.7, I42.9, I11.0, I13.0, I13.2) at the Heart Centre or the Department of Internal medicine at Umeå University Hospital, Sweden, between January 2010 and December 2019 and who lived within the catchment area of the hospital. The diagnosis could be from either a HF hospitalisation event or outpatient visit and includes both prevalent cases and new onset HF during the inclusion period. The hospital is the only one within its geographic catchment area and serves a mixed urban and rural population of about 150 000 people and has the only specialist cardiology clinic in the area.

### Data collection

The patients were identified in the hospitals electronic health records system (NCS Cross). Clinical parameters were extracted from the patients’ electronic health records using a prespecified protocol encompassing lab results, echocardiogram data, ECG results, comorbidities, medication and known diabetes status. DM was categorised as either Type 1 DM (T1DM) or T2DM, according to ICD 10 codes. Coronary artery disease was defined as either a previous myocardial infarction or the presence of at least one stenosis of 50% or more in a major coronary vessel. The latest available value for each parameter was used regardless of if it was from an inpatient or outpatient setting, including echocardiogram results. HF phenotype was categorised according to left ventricle EF (LVEF) as HF with reduced EF (HFrEF, EF ≤40), HF with mildly reduced ejection (HFmrEF, EF 41%–49%) or HFpEF (EF ≥50%).^17^ Lab, ECG and echocardiogram data were available regardless of which department had ordered them, including primary care. New York Heart Association (NYHA) class was not routinely stated in patient files and could not be assessed.

### Diabetes and pre-diabetes classification

Data on HbA1c and fasting plasma-glucose (FPG) were recorded if there was a value from within 1 year from the otherwise latest available lab result. It was not possible to ascertain in every case if a plasma glucose measurement was fasting or not. Due to this uncertainty, we put more emphasis on HbA1c levels when classifying patients’ DM status. With that in mind, we categorised patients’ DM status using the following criteria, based on WHO recommendations^18 19^:

- Known DM: Presence of a clinical diagnosis of DM.
- Probable DM: HbA1c≥48 mmol/mol, or FPG≥7.0 mmol/L in the absence of a HbA1c-value.
- Pre-diabetes:
  - Impaired fasting glucose (IFG) defined as FPG≥6.1 mmol/L if HbA1c <48 mmol/mol, or FPG 6.1–6.9 mmol/L in the absence of a HbA1c value.
  - Intermediate hyperglycaemia defined as HbA1c 42–47 mmol/mol.
- Non-DM: No previous diagnosis of DM and not meeting criteria for probable DM or pre-diabetes.
- Unknown: No previous diagnosis of DM and no glycaemic tests available.

### Statistical analysis

Extracted data on patients who were alive 1 January 2020 were analysed using SPSS V.27. Continuous parameters are presented as means with SDs. Continuous variables without normal distribution are presented as medians with IQR. Categorical variables are presented with frequencies and percentage. When analysing differences between glycaemic profiles, each group was compared with the non-DM group using Pearson’s χ^2^ test. When analysing differences between HF phenotypes, a test of homogeneity was performed between all groups. If that was significant, pairwise comparisons between groups were made. The null hypothesis used was that there are no significant differences between groups. If a group had less than five patients, Fisher’s exact test was used instead. A p<0.05 was considered significant.

## Results

A total of 2433 patients with a diagnosis of HF were alive 1 January 2020. Of these, 107 did not have an echocardiogram recorded and no EF available and were excluded. Of the 2326 patients with known EF, a majority (59%) were men, 617 (27%) had known DM, 26 with T1DM and 591 with T2DM. A total of 1084 patients (47%) had HFpEF, 561 patients (24%) had HFmrEF and 681 patients (29%) HFrEF. There were differences in many clinical characteristics between men and women. See table 1 for clinical characteristics for the cohort as a whole as well as for men and women, respectively.

**Table 1:** Clinical characteristics

Characteristics*	Total N=2326	Men N=1363 (59%)	Women N=963 (41%)	P value men versus women
Age—year	76±13	74±12	78±12	<0.001
Blood pressure—mm Hg				
Systolic	128±20	127±19	130±21	<0.001
Diastolic	74±11	75±11	73±12	<0.001
Heart rate—beats/min	74±16	72±15	75±16	<0.001
Median NT-pro-BNP—ng/L	874 (292–2101)	793 (265–2042)	986 (330–2202)	0.006
Left ventricle ejection fraction— %	47±11	45±11	50±11	<0.001
>50%—no (%)	1084 (47)	536 (39)	548 (57)	<0.001
41%–49%—no (%)	561 (24)	354 (26)	207 (22)	0.015
≤40% no (%)	681 (29)	473 (35)	208 (22)	<0.001
eGFR—mL/min/1.73 m^2^	56±20	58±20	53±19	<0.001
Body mass index—kg/m^2^	28±6	28±5	28±6	0.34
Medical history—no (%)				
Atrial fibrillation	1129 (52)	675 (50)	454 (47)	0.28
Diabetes mellitus	617 (27)	387 (28)	230 (24)	0.017
Hypertension	1677 (76)	971 (71)	706 (73)	0.29
Coronary disease*	922 (40)	631 (46)	291 (30)	<0.001
Hospitalisation for HF	915 (39)	525 (39)	390 (41)	0.36
HF treatments—no (%)				
ACE inhibitor or ARB	1831 (79)	1068 (78)	763 (79)	0.65
Beta-blocker	1967 (85)	1157 (85)	810 (84)	0.65
MRA	1039 (45)	653 (48)	386 (40)	<0.001
ARNI	148 (6)	122 (9)	26 (3)	<0.001
Loop diuretics	1272 (55)	695 (51)	577 (60)	<0.001
SGLT2-inhibitor	78 (3)	62 (5)	16 (2)	<0.001
Digitalis	250 (11)	134 (10)	116 (12)	0.1
ICD†	185 (8)	149 (11)	36 (4)	<0.001
CRT†	195 (8)	142 (10)	53 (6)	<0.001
Glycaemic status				
Unknown	617 (27)	349 (26)	268 (28)	0.23
Normoglycaemic	548 (24)	315 (23)	233 (24)	0.55
Pre-diabetes	447 (19)	264 (19)	183 (19)	0.83
Probable DM	97 (4)	48 (4)	49 (5)	0.07
DM	617 (27)	387 (28)	230 (24)	0.017
Type 1	26	14 (1)	12 (1)	
Type 2	591	373 (27)	218 (23)	

Clinical characteristics of heart failure patients with known ejection fraction in the Umeå Cohort. P values are from comparisons between men and women.

Unknown; no available glycaemic values for analysis.

*Coronary artery disease defined as either previous MI or documented stenosis of at least 50%.

†Including patients with CRT defibrillator.

ACE, angiotensin converting enzyme; ARB, angiotensin receptor blocker; ARNI, angiotensin receptor blocker-neprilysin inhibitor; CRT, cardiac resynchronisation therapy; DM, diabetes mellitus; eGFR, estimated glomerural filtration rate; HF, heart failure; ICD, implantable cardioverter-defibrillator; MRA, mineralocorticoid receptor blocker; NT-pro-BNP, N-terminal pro-B-type natriuretic peptide SGLT2, sodium-glucose cotransporter 2.

**Figure 1:**
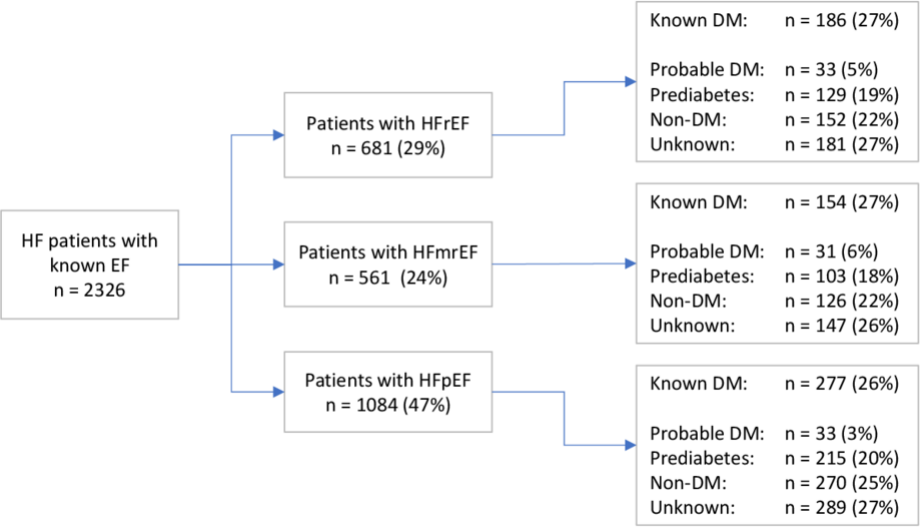
Distribution of patients according to heart failure classification and diabetes status. There were no statistically significant differences (p=0.32) in distribution of diabetes status between heart failure phenotypes. HFrEF, heart failure with reduced ejection fraction; HFmrEF, heart failure with mildly reduced ejection fraction; HFpEF, heart failure with preserved ejection fraction; known DM, patients with a diagnosis of diabetes mellitus; probable DM, patients with glycaemic test results at diabetic levels; non-DM, patients without a diagnosis of diabetes mellitus and normal glycaemic results; unknown, patients without available glycaemic data.

Of the 1709 patients without DM, 577 (34%) had both a HbA1c and an FPG recorded within the inclusion period, while 274 (16%) patients had an FPG but no HbA1c, and 247 (14%) patients had a HbA1c but no FPG recorded. 617 (36%) of the patients without previously known DM had neither an FPG nor HbA1c recorded. When applying the stated criteria for DM status on the 1092 patients without previous DM diagnosis with available FPG or HbA1c tests, 97 (9%) patients were deemed to have probable DM and 447 (41%) patients pre-diabetes (of which 216 IFG, 322 intermediate hyperglycaemia and 91 patients both). DM status did not differ significantly between HF phenotype (p=0.32, figure 1). Clinical characteristics of patients by DM status are available in online supplemental file 1.

10.1136/openhrt-2022-002133.supp1 Supplementary data

Non-DM patients were less often treated with beta blockers and loop diuretics compared with patients with pre-diabetes or known DM and tended to be less often treated with ACE inhibitors or angiotensin receptor blocker. Patients with probable DM had the highest rate of loop diuretic and MRA treatment. There were no differences between groups regarding treatment with angiotensin receptor blocker-neprilysin inhibitor (ARNI) or digitalis. Sodium-glucose cotransporter 2 (SGLT2)-inhibitor treatment only occurred in probable DM (1%) and known DM (12%) (table 2).

**Table 2:** Heart failure treatment according to diabetes status

Treatment	Non-DM (N=548)	Pre-diabetes (N=447)		P value (vs non-DM)	Probable DM (N=97)	P value (vs non-DM)	Known DM (N=617)	P value (vs non-DM)
ACE inhibitor or ARB	427 (78)	366 (82)	0.007		81 (84)	0.27	507 (82)	0.69
Beta-blocker	453 (83)	390 (87)	0.006		81 (84)	0.60	544 (88)	<0.001
Loop diuretics	230 (42)	250 (56)	<0.001		70 (72)	<0.001	391 (64)	<0.001
MRA	235 (43)	181 (41)	0.49		53 (55)	0.042	294 (48)	0.10
ARNI	36 (7)	21 (5)	0.26		3 (3)	0.27	38 (6)	0.87
SGLT2 inhibitors	0 (0)	0 (0)	–		1 (1)	0.15	75 (12)	<0.001
Digitalis	49 (9)	51 (11)	0.24		12 (12)	0.38	71 (12)	0.18
ICD†	54 (10)	26 (6)	0.030		2 (2)	0.025	56 (9)	0.77
CRT†	23 (4)	20 (5)	1.0		8 (8)	0.21	33 (5)	0.45

Comparison of heart failure treatment between normoglycaemic patients with patients and pre-diabetes, probable diabetes mellitus and known diabetes mellitus, respectively.

*Statistical significance level p<0.05. Comparisons are between the non-DM and each other group, respectively.

†Including CRT defibrillator.

ACE, angiotensin converting enzyme; ARB, angiotensin receptor blocker; ARNI, angiotensin receptor blocker-neprilysin inhibitor; CRT, cardiac resynchronisation therapy; DM, diabetes mellitus; ICD, implantable cardioverter-defibrillator; MRA, mineralocorticoid receptor blocker; SGLT2, sodium-glucose cotransporter 2.

Of the 617 patients with known diabetes, 301 were treated with metformin, 245 with insulin, 106 with dipeptidyl peptidase four inhibitors, 75 with SGLT2 inhibitors, 47 with glucagon-like peptide one receptor analogues and 38 with sulphonylureas (including repaglinide). There were no differences in choice of DM treatment across HF phenotypes (table 3).

**Table 3:** Diabetes treatment according to HF phenotype

Diabetes medication	HFrEF (n=186)	HFmrEF (n=154)	HFpEF (n=277)	P value
SGLT2-inhibitor—no (%)	29 (16)	18 (12)	28 (10)	0.20
Metformin—no (%)	84 (45)	75 (49)	142 (51)	0.43
Insulin—no (%)	80 (43)	54 (35)	111 (40)	0.32
DPP4-inhibitor—no (%)	22 (12)	33 (21)	51 (18)	0.050
Sulfonylurea or repaglinid—no (%)	17 (9)	7^5^	14 (5)	0.14
GLP1 receptor agonists—no (%)	14 (8)	13 (9)	20 (7)	0.90

Comparison of prescribed diabetes drugs according to heart failure phenotype to 617 patients with known DM.

P value calculated with χ^2^ test of homogeneity.

DM, diabetes mellitus; DPP4, dipeptidyl peptidase 4; GLP1, glucagon-like peptide 1; HFmrEF, heart failure with mildly reduced ejection fraction; HFpEF, heart failure with preserved ejection fraction; HFrEF, Heart failure with reduced ejection fraction; SGLT2, sodium-glucose cotransporter 2.

## Discussion

In this real-world HF population, one-fourth of patients had previously known DM. Of the patients without previous DM with available glycaemic tests, 1/10 had probable DM and 4/10 pre-diabetes. Diabetes and pre-diabetes prevalence and treatment did not differ between HF phenotypes. DM and pre-diabetes patients tended to have higher rates of diuretics and beta blockers prescribed.

There are three ways to diagnose pre-diabetes and diabetes—FPG, HbA1c and through an oral glucose tolerance test (OGTT).^20^ An OGTT is the most sensitive and specific test,^21 22^ but is not routinely used due to being more time- and resource intensive than the alternatives. FPG has the lowest sensitivity but good specificity. HbA1c has low specificity and sensitivity compared with an OGTT, but higher sensitivity than FPG.^21^ In this study, we have used both FPG and HbA1c-levels to characterise patients’ glycaemic status. The two measurements were available in roughly equal number of patients, but more patients were found to have pre-diabetes with HbA1c than with FPG, indicating that it is more sensitive than FPG in this population as well. Patients who were identified with each method were only partially overlapping. Previous prevalence estimates for pre-diabetes in HF patients have mainly been based on HbA1c levels alone^10 11^ or FPG alone,^9 23^ and have been performed in different settings. The work of Gotsman *et al*^9^ is the most similar to this study, but only used FPG to diagnose pre-diabetes. The proportion of known DM was higher than in our study (49.5% vs 27%), but the proportion of pre-diabetes was lower (12% vs 19%). One reason for that discrepancy could be that the prevalence of pre-diabetes is underestimated if only FPG or HbA1c is used. A generous approach to testing with FPG and HbA1c in HF patients would likely identify more cases of pre-diabetes, with the addition of an OGTT in selected patients.

Patients with known DM and pre-diabetes were more often treated with beta blockers and diuretics compared with non-DM patients. This might indicate that these patients have more symptomatic HF compared with non-DM patients, which would be in line with the worse prognosis seen in HF patients with DM.^17 24^

The usage of SGLT2-inhibitors was generally low, at only 3%. This reflects that the data used for this study covers the time up to December 2019, which was before SGLT2-inhibitors were approved for HF treatment without DM. If this study was repeated today, SGLT2 inhibitor treatment rates would likely be markedly higher.

Use of ARNI was low at only 6% of all patients, corresponding to 22% of the number of HFrEF patients, but did not differ between glycaemic groups. The prescription rate in our healthcare region was still above average compared with national rates,^25^ and in line with international findings^26 27^ that ARNI implementation is slow. There could be many reasons for low ARNI uptake, including clinical inertia, strict prescription criteria and patient factors. Old age and renal impairment have been implicated as reasons for low usage of ARNI and other guideline directed medical treatment,^28^ and our cohort has those factors compared with drug trial populations.^15 16^ There have been efforts to increase use of ARNI and other HF therapies with targeted programmes,^29^ including at our centre,^30^ but still only a minority of patients receive ARNI.

Our cohort was reasonably balanced in regard to sex, with 59% men and 41% women. However, women were significantly older with more HFpEF, less HFrEF and worse renal function, and less use of MRA, ARNI and SGLT2-inhibitors, which is commonly seen in HF cohorts.^31^ In this cohort, lower use of medications recommended for HFrEF could partly be explained by a lower rate of HFrEF, and lower use of SGLT2-inhibitors due a lower rate of DM, but women are often undertreated in HF studies.^31^ A previous study by Norberg *et al*^32^ in this cohort found that the main determinants for lower medication rate was older age, worse renal function and lower body weight, and not sex in and of itself.

While pre-diabetes is a risk factor for adverse outcomes in HF, the benefit of identifying and treating pre-diabetes in HF is unclear. Treatment of other comorbidities in HF patients, including DM, is considered important, and choice of DM therapy should be influenced by the presence of HF.^17 20^ Specifically, SGLT2-inhibitors are effective both in the treatment of DM and HF (regardless of LVEF or DM status), as well as chronic kidney disease,^14 17 20 33^ which are all conditions with high overlap. Considering the well-established effects and strong recommendation for SGLT2-inhibitors in HF,^34^ many patients with concomitant pre-diabetes and DM, both known and unknown, will receive SGLT2-inhibitor treatment as an added benefit. This extra glucose-lowering effect could necessitate adjustment in other glucose-lowering therapy. Due to high overlap with ischaemic heart disease and HF, identification of DM is important in HF patients so that treatment can be individualised with, for example, GLP1 receptor agonists in eligible patients.^35^ Since pre-diabetes and probable DM is common in HF patients, it is reasonable to have a generous approach to glycaemic testing in HF patients.

There is evidence for lifestyle and drug interventions in pre-diabetes to prevent progression to T2DM and to lower the risk of cardiovascular disease,^36 37^ but there is lacking evidence if the same interventions are effective against pre-diabetes in patients with established HF, and what the clinical benefit of those interventions are. This should be an avenue of further research.

## Limitations

This study is limited by being a single-centre study based on data from patient records, so the results might not be generalisable to other populations or settings. The wide patient selection criteria used has both advantages and drawbacks. The intent and the advantage of this method is that as many HF patients as possible could be included so that results are applicable to the full span of HF patients encountered in the clinic. The drawbacks include that the patient cohort is heterogeneous with regard to HF type, aetiology, duration and clinical setting, which makes the results less applicable to specific HF subpopulations. Using retrospective data from patient files meant that we did not have complete or current data on all patients. Specifically, FPG and HbA1c were missing on many patients. Additionally, some recorded FPG values could be from non-fasting patients due to missing or erroneous labelling in patient files, which is why we relied more on HbA1c levels than FPG to classify a patient as having probable DM. Of patients without previous DM with available tests, half had some level of dysglycaemia. The rate in the whole population might be lower due to bias in which patients were tested, but our results indicate that blood glucose abnormalities are very common, despite limitations. Prevalence numbers are also affected by which criteria are used. The American Diabetes Association (ADA) recommend lower limits than the WHO,^20 38^ so more patients would have been classified as having pre-diabetes if we had used the ADA criteria.

NYHA class was another parameter that was rarely stated in patient files. This limits us from analysing our material regarding symptomatic severity and how it correlated to glycaemic class and choice of treatment.

Another limitation is that we used the latest available echocardiogram to classify patients’ HF phenotype. Patients with HF with recovered EF would therefore be classified as HFmrEF or HFpEF, instead of HFrEF as the European Society of Cardiology guidelines recommend.^17^

## Conclusions

In this Northern European real-world HF population, the prevalence of known DM was 27%. Of patients without known DM with available FPG or HbA1c-measurements, 9% had probable DM and 41% pre-diabetes, corresponding to 4% and 19% of the entire cohort, respectively. The prevalence of DM, pre-diabetes or normoglycaemia or choice of DM treatment did not differ between HF phenotypes. Patients with pre-diabetes and DM were more often treated with diuretics and beta blockers than normoglycaemic patients.

## Data Availability

Data are available on reasonable request. Request should be made to the corresponding author. Data is available in the form of deidentified spreadsheets.

## References

[1] Boman K, Lindmark K, Stålhammar J, et al. Healthcare resource utilisation and costs associated with a heart failure diagnosis: a retrospective, population-based cohort study in Sweden. BMJ Open 2021;11:e053806. 10.1136/bmjopen-2021-053806 PMC8527145 34667015

[2] Khan MAB, Hashim MJ, King JK, et al. Epidemiology of type 2 diabetes – global burden of disease and Forecasted trends. J Epidemiol Glob Health 2019;10:107. 10.2991/jegh.k.191028.001 PMC7310804 32175717

[3] Maack C, Lehrke M, Backs J, et al. Heart failure and diabetes: metabolic alterations and therapeutic interventions: a state-of-the-art review from the translational research Committee of the heart failure Association-European Society of cardiology. Eur Heart J 2018;39:4243–54. 10.1093/eurheartj/ehy596 30295797 PMC6302261

[4] Mentz RJ, Kelly JP, von Lueder TG, et al. Noncardiac comorbidities in heart failure with reduced versus preserved ejection fraction. J Am Coll Cardiol 2014;64:2281–93. 10.1016/j.jacc.2014.08.036 25456761 PMC4254505

[5] Ambrosy AP, Fonarow GC, Butler J, et al. The global health and economic burden of hospitalizations for heart failure: lessons learned from hospitalized heart failure registries. J Am Coll Cardiol 2014;63:1123–33. 10.1016/j.jacc.2013.11.053 24491689

[6] McHugh K, DeVore AD, Wu J, et al. Heart Failure With Preserved Ejection Fraction and Diabetes: JACC State-of-the-Art Review. J Am Coll Cardiol 2019;73:602–11. 10.1016/j.jacc.2018.11.033 30732715

[7] CommitteeIE, International Expert Committee. International expert Committee report on the role of the A1c assay in the diagnosis of diabetes. Diabetes Care 2009;32:1327–34. 10.2337/dc09-9033 19502545 PMC2699715

[8] Dunlay SM, Givertz MM, Aguilar D, et al. Type 2 diabetes mellitus and heart failure, a scientific statement from the American heart association and heart failure Society of America. J Card Fail 2019;25:584–619. 10.1016/j.cardfail.2019.05.007 31174952

[9] Gotsman I, Shauer A, Lotan C, et al. Impaired fasting glucose: a predictor of reduced survival in patients with heart failure. Eur J Heart Fail 2014;16:1190–8. 10.1002/ejhf.146 25080892

[10] Kristensen SL, Jhund PS, Lee MMY, et al. Prevalence of prediabetes and undiagnosed diabetes in patients with HFpEF and HFrEF and associated clinical outcomes. Cardiovasc Drugs Ther 2017;31:545–9. 10.1007/s10557-017-6754-x 28948430 PMC5730631

[11] Jackson AM, Rørth R, Liu J. Diabetes and pre-diabetes in patients with heart failure and preserved ejection fraction. Eur J Heart Fail. 2021. 10.1002/ejhf.2403 PMC9542636 34918855

[12] Kristensen SL, Preiss D, Jhund PS, et al. Risk related to pre-diabetes mellitus and diabetes mellitus in heart failure with reduced ejection fraction: insights from prospective comparison of ARNI with ACEI to determine impact on global mortality and morbidity in heart failure trial. Circ Heart Fail 2016;9. 10.1161/CIRCHEARTFAILURE.115.002560 PMC4718182 26754626

[13] McMurray JJV, Solomon SD, Inzucchi SE, et al. Dapagliflozin in patients with heart failure and reduced ejection fraction. N Engl J Med 2019;381:1995–2008. 10.1056/NEJMoa1911303 31535829

[14] Packer M, Anker SD, Butler J, et al. Cardiovascular and renal outcomes with Empagliflozin in heart failure. N Engl J Med 2020;383:1413–24. 10.1056/NEJMoa2022190 32865377

[15] Norberg H, Bergdahl E, Lindmark K. Eligibility of sacubitril-valsartan in a real-world heart failure population: a community-based single-centre study. ESC Heart Fail 2018;5:337–43. 10.1002/ehf2.12251 29345425 PMC5880656

[16] Håkansson E, Norberg H, Själander S, et al. Eligibility of dapagliflozin and Empagliflozin in a real-world heart failure population. Cardiovasc Ther 2021;2021:1–8. 10.1155/2021/1894155 PMC8720587 35024052

[17] McDonagh TA, Metra M, Adamo M, et al. 2021 ESC guidelines for the diagnosis and treatment of acute and chronic heart failure. Eur Heart J 2021;42:3599–726. 10.1093/eurheartj/ehab368 34447992

[18] Diagnosis and management of type 2 diabetes (HEARTS-D) 2020 Geneva World Health organization

[19] WHO. Definition and diagnosis of diabetes mellitus and intermediate hyperglycaemia : report of a WHO/IDF consultation. World Health Organization & International Diabetes Federation, 2006. https://apps.who.int/iris/handle/10665/43588

[20] Cosentino F, Grant PJ, Aboyans V, et al. 2019 ESC guidelines on diabetes, pre-diabetes, and cardiovascular diseases developed in collaboration with the EASD. Eur Heart J 2020;41:255–323. 10.1093/eurheartj/ehz486 31497854

[21] Barry E, Roberts S, Oke J, et al. Efficacy and effectiveness of screen and treat policies in prevention of type 2 diabetes: systematic review and meta-analysis of screening tests and interventions. BMJ 2017;356:i6538. 10.1136/bmj.i6538 28052845

[22] Bartnik M, Rydén L, Malmberg K, et al. Oral glucose tolerance test is needed for appropriate classification of glucose regulation in patients with coronary artery disease: a report from the Euro heart survey on diabetes and the heart. Heart 2007;93:72–7. 10.1136/hrt.2005.086975 16905628 PMC1861359

[23] Chen Y-Y, Chen Y, Liang S-M, et al. Prognostic impact of fasting plasma glucose on mortality and Re-Hospitalization in patients with acute heart failure. Chin Med J 2018;131:2032–40. 10.4103/0366-6999.239310 30127212 PMC6111696

[24] From AM, Leibson CL, Bursi F, et al. Diabetes in heart failure: prevalence and impact on outcome in the population. Am J Med 2006;119:591–9. 10.1016/j.amjmed.2006.05.024 16828631

[25] Fu M, Vedin O, Svennblad B, et al. Implementation of sacubitril/valsartan in Sweden: clinical characteristics, titration patterns, and determinants. ESC Heart Fail 2020;7:3633–43. 10.1002/ehf2.12883 32881399 PMC7754959

[26] Savarese G, Bodegard J, Norhammar A, et al. Heart failure drug titration, discontinuation, mortality and heart failure hospitalization risk: a multinational observational study (US, UK and Sweden). Eur J Heart Fail 2021;23:1499–511. 10.1002/ejhf.2271 34132001

[27] Greene SJ, Butler J, Albert NM, et al. Medical Therapy for Heart Failure With Reduced Ejection Fraction: The CHAMP-HF Registry. J Am Coll Cardiol 2018;72:351–66. 10.1016/j.jacc.2018.04.070 30025570

[28] Greene SJ, Tan X, Yeh Y-C, et al. Factors associated with non-use and sub-target dosing of medical therapy for heart failure with reduced ejection fraction. Heart Fail Rev 2022;27:741–53. 10.1007/s10741-021-10077-x 33471236

[29] Gulizia MM, Orso F, Mortara A, et al. BLITZ-HF: a nationwide initiative to evaluate and improve adherence to acute and chronic heart failure guidelines. Eur J Heart Fail 2022. doi:10.1002/ejhf.2605. [Epub ahead of print: 03 Jul 2022]. PMC10084144 35785461

[30] Norberg H, Bergdahl E, Ängerud KH, et al. A systematic approach for introduction of novel treatments to a chronic patient group: sacubitril-valsartan as a case study. Eur J Clin Pharmacol 2021;77:125–31. 10.1007/s00228-020-02979-w 32820363 PMC7782406

[31] Galati G, Sabouret P, Germanova O, et al. Women and diabetes: preventing heart disease in a new era of therapies. Eur Cardiol 2021;16:e40. 10.15420/ecr.2021.22 34777580 PMC8576483

[32] Norberg H, Pranic V, Bergdahl E, et al. Differences in medical treatment and clinical characteristics between men and women with heart failure - a single-centre multivariable analysis. Eur J Clin Pharmacol 2020;76:539–46. 10.1007/s00228-019-02782-2 31897534

[33] Heerspink HJL, Stefánsson BV, Correa-Rotter R, et al. Dapagliflozin in patients with chronic kidney disease. N Engl J Med 2020;383:1436–46. 10.1056/NEJMoa2024816 32970396

[34] Vaduganathan M, Docherty KF, Claggett BL, et al. SGLT-2 inhibitors in patients with heart failure: a comprehensive meta-analysis of five randomised controlled trials. Lancet 2022;400:757–67. 10.1016/S0140-6736(22)01429-5 36041474

[35] Sabouret P, Galati G, Angoulvant D, et al. The interplay between cardiology and diabetology: a renewed collaboration to optimize cardiovascular prevention and heart failure management. Eur Heart J Cardiovasc Pharmacother 2020;6:394–404. 10.1093/ehjcvp/pvaa051 32402065

[36] Gong Q, Zhang P, Wang J, et al. Morbidity and mortality after lifestyle intervention for people with impaired glucose tolerance: 30-year results of the dA Qing diabetes prevention outcome study. Lancet Diabetes Endocrinol 2019;7:452–61. 10.1016/S2213-8587(19)30093-2 31036503 PMC8172050

[37] Khetan AK, Rajagopalan S. Prediabetes. Can J Cardiol 2018;34:615–23. 10.1016/j.cjca.2017.12.030 29731022

[38] Association AD. 2. classification and diagnosis of diabetes. Diabetes Care 2021;44:S15–33. 33298413 10.2337/dc21-S002

