# Supplementary material for: Prevalence and treatment of diabetes and pre-diabetes in a real-world heart failure population: a single-centre cross-sectional study
Source: Open Heart. 2022 Dec 13;9(2):e002133. doi: 10.1136/openhrt-2022-002133 (PMC9748948; doi:10.1136/openhrt-2022-002133)
Supplement: Supplementary data [file openhrt-2022-002133supp001.pdf]

Supplement 1

Characteristics*	Unknown (N=617)	Non-DM (N=548)	Prediabetes (N=447)	Probable DM (N=97)	Known DM (N=617)
Age - yr	74.8 ± 15.1	74.3 ± 13.1	78.3 ± 10.6	82.9 ± 10.3	76.5 ± 9.7
Female sex - no. (%)	268 (43)	233 (43)	183 (41)	49 (51)	229 (37)
Systolic blood pressure - mmHg	126 ± 20	127 ± 20	130 ± 19	129 ± 22	130 ± 20
Diastolic blood pressure - mmHg	73 ± 11	75 ± 12	74 ± 11	72 ± 14	73 ± 11
Median NTproBNP - pg/ml	889 (289 - 2010)	812 (269-1941)	911 (294-2026)	1696 (575-3095)	904 (320-2387)
Creatinine clearance - ml/min	58 ± 20	59 ± 19	54 ± 17	47 ± 18	53 ± 21
Heart rate - beats/min	74 ± 16	72 ± 15	72 ± 17	75 ± 14	76 ± 16
Body-mass index - kg/m^2	26.7 ± 5.3	26.7 ± 5.2	27.9 ± 5.6	27.5 ± 5.8	30.1 ± 5.9
Ejection fraction (%)	47 ± 11	48 ± 11	48 ± 11	45 ± 11	47 ± 11
**Medical history - no. (%)**					
Atrial fibrillation	306 (50)	248 (45)	213 (48)	60 (62)	301 (49)
Coronary artery disease[a]	153 (25)	194 (36)	206 (46)	54 (56)	315 (51)
Hypertension	370 (60)	365 (67)	341 (76)	79 (81)	521 (85)
Hospitalized for HF	240 (39)	191 (35)	175 (39)	46 (47)	263 (43)

Comparsion of patient characteristics, comorbidities and heart failure treatment between normoglycaemic patients with patients and prediabetes, probable diabetes mellitus and known diabetes mellitus, respectively. *Values are means ±SD, %, or median (interquartile range). a Coronary artery disease definied as either previous MI or documented stenosis of at least 50 %.
